# Inhibition of MEK1 Signaling Pathway in the Liver Ameliorates Insulin Resistance

**DOI:** 10.1155/2016/8264830

**Published:** 2015-12-28

**Authors:** Atsunori Ueyama, Nobuhiro Ban, Masanori Fukazawa, Tohru Hirayama, Minako Takeda, Tatsuo Yata, Hiroyasu Muramatsu, Masaki Hoshino, Marii Yamamoto, Masao Matsuo, Yuka Kawashima, Tatsuhiko Iwase, Takehisa Kitazawa, Youichi Kushima, Yuichiro Yamada, Yoshiki Kawabe

**Affiliations:** ^1^Research Division, Chugai Pharmaceutical Co., Ltd., 1-135 Komakado, Gotemba, Shizuoka 412-8513, Japan; ^2^Department of Endocrinology, Diabetes and Geriatric Medicine, Akita University School of Medicine, 1-1-1 Hondo, Akita, Akita 010-8543, Japan; ^3^Chugai Research Institute for Medical Science, 1-135 Komakado, Gotemba, Shizuoka 412-8513, Japan; ^4^Project Planning & Coordination Department, Chugai Pharmaceutical Co., Ltd., 2-1-1 Nihonbashi, Muromachi, Chuo-ku, Tokyo 103-8324, Japan

## Abstract

Although mitogen-activated protein kinase kinase (MEK) is a key signaling molecule and a negative regulator of insulin action, it is still uncertain whether MEK can be a therapeutic target for amelioration of insulin resistance (IR) in type 2 diabetes (T2D) *in vivo*. To clarify whether MEK inhibition improves T2D, we examined the effect of continuous MEK inhibition with two structurally different MEK inhibitors, RO5126766 and RO4987655, in mouse models of T2D. RO5126766 and RO4987655 were administered via dietary admixture. Both compounds decreased blood glucose and improved glucose tolerance in doses sufficient to sustain inhibition of extracellular signal-regulated kinase (ERK)1/2 phosphorylation downstream of MEK in insulin-responsive tissues in *db/db* mice. A hyperinsulinemic-euglycemic clamp test showed increased glucose infusion rate (GIR) in *db/db* mice treated with these compounds, and about 60% of the increase was attributed to the inhibition of endogenous glucose production, suggesting that the liver is responsible for the improvement of IR. By means of adenovirus-mediated *Mek1* shRNA expression, we confirmed that blood glucose levels are reduced by suppression of MEK1 expression in the liver of *db/db* mice. Taken together, these results suggested that the MEK signaling pathway could be a novel therapeutic target for novel antidiabetic agents.

## 1. Introduction

The pathology of type 2 diabetes (T2D) is characterized by impaired insulin secretion from pancreatic beta cells and impaired insulin action, known as insulin resistance (IR). Although glucagon-like peptide-1 receptor agonists and sodium-glucose cotransporter 2 inhibitors [1, 2] have become available to treat T2D, currently the only clinically available insulin sensitizers are peroxisome proliferator-activated receptor (PPAR)*γ* agonists, such as pioglitazone.

Insulin initiates the regulation of various cell functions through the phosphoinositide 3-kinase (PI 3-K) pathway and the mitogen-activated protein kinase kinase (MEK) pathway after binding to insulin receptors and becoming phosphorylated [3, 4]. It is believed that the PI 3-K pathway is important in glucose metabolism [5], whereas the MEK pathway is considered to mainly control cell growth and differentiation [6, 7]; however, the precise role of MEK in the regulation of glucose metabolism by insulin is still not fully established. There are several lines of* in vitro* evidence suggesting that the MEK pathway negatively regulates insulin action: (i) activated extracellular signal-regulated kinase (ERK) phosphorylates IRS-1 Ser^307^ residue and impairs insulin signal transduction [5, 8–11]; (ii) MEK inhibition leads to increased protein kinase B (Akt) phosphorylation and to improved insulin signaling accompanied by the reduction in IRS-1 Ser^307^ phosphorylation [12]; and (iii) MEK1 is a major kinase involved in inducing IR in 3T3-L1 adipocytes [11]. On the other hand, it has been reported that the constitutive active form of MEK expressed in the liver has insulinotropic effects without altering insulin sensitivity* in vivo* [13]. From gene knockout studies in mice, it has been reported that ERK1 knockout mice are fertile and of normal size and have defective T cell differentiation, enhanced long-term memory, and potentiation in the nucleus accumbens, decreased adiposity, and improved insulin sensitivity [14–17]. On the other hand, MEK1 or ERK2 knockout mice are embryonic lethal [18–21]. Therefore, it is unclear whether MEK is an appropriate therapeutic target for ameliorating insulin resistance in T2D.

Two novel MEK inhibitors (RO4987655 and RO5126766) are currently under clinical development for cancer treatment. A Phase 1 dose escalation study of RO4987655, a pure MEK inhibitor, has been completed [22] and an expansion study is ongoing [23]. Phase 1 studies of RO5126766, a dual Raf/MEK inhibitor [24, 25], have been completed in both Japan and Europe [26, 27], and an alternative study is ongoing.

In the present study, we describe the antidiabetic effect of MEK inhibition with these MEK inhibitors in* db/db* mice, a T2D animal model, and explore the mechanism underlying the glucose lowering effect of MEK1 inhibition.

## 2. Materials and Methods

### 2.1. Materials

**Figure 1:**
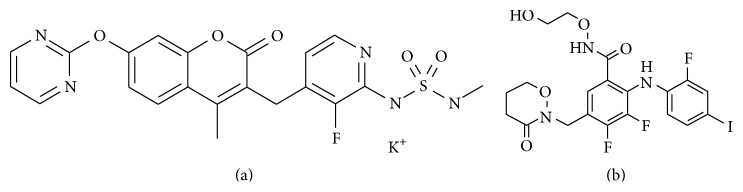
Chemical structures of (a) RO5126766 (dual Raf/MEK inhibitor) and (b) RO4987655 (specific MEK1/2 inhibitor).

RO5126766 (3-[[3-fluoro-2-(methylsulfamoylamino)-4-pyridyl]methyl]-4-methyl-7-pyrimidin-2-yloxychromen-2-one) (Figure 1(a)) [24–26] and RO4987655 (3,4-difluoro-2-((2-fluoro-4-iodophenyl)amino)-N-(2-hydroxyethoxy)-5-((3-oxo-1,2-oxazinan-2-yl)methyl)benzamide) (Figure 1(b)) [22, 28] were synthesized in our laboratories at Chugai Pharmaceutical Co., Ltd. Pioglitazone hydrochloride was purchased from Tokyo Chemical Industry Co., Ltd. (Tokyo, Japan). Insulin (Novolin R; 100 IU·mL^−1^) was purchased from Novo Nordisk Pharma (Tokyo, Japan). Uniformly labeled [U-^13^C]glucose (99-atom percent excess) was purchased from Cambridge Isotope Laboratories (Andover, MA, USA). Glucose solution (50%) was purchased from Otsuka Pharmaceutical Factory Inc. (Tokushima, Japan). Glucose solution (50%) was diluted with purified water to make concentrations of 20% for the oral glucose tolerance test (OGTT) and 10% for the hyperinsulinemic-euglycemic clamp test. Insulin ELISA kit was purchased from Morinaga Institute of Biological Science, Inc. (Yokohama, Japan). RIPA buffer, Halt protease inhibitor cocktail (100x), Halt phosphatase inhibitor cocktail (100x), and SuperSignal West Femto Maximum Sensitivity Substrate were purchased from Thermo Fisher Scientific (Rockford, IL, USA). Polyvinylidene difluoride membrane (PVDF) (0.2 *µ*m) was purchased from Bio-Rad Laboratories Inc. (Hercules, CA, USA).

### 2.2. Preparation of Dietary Admixtures

High-concentration stock solutions of RO5126766 or RO4987655 were made by dissolving the compound in water or ethanol, respectively. After dilution to 3.44 mg·50 mL^−1^ (RO5126766) and 8 mg·50 mL^−1^ (RO4987655) with the same vehicle, they were added little by little to 1 kg of laboratory chow (CE-2 powder; CLEA Japan, Tokyo, Japan) and mixed using a food processor. The mixtures were then dried with a vacuum pump for at least overnight to remove the vehicle. Dietary admixtures comprising several different dosages of RO5126766 or RO4987655 were made in this way and stored at −30°C until use.

### 2.3. Animals

All animal care and experiments were performed in accordance with the guidelines for the care and use of laboratory animals at Chugai Pharmaceutical Co., Ltd., and all protocols were approved by the Institutional Animal Care and Use Committee at Chugai Pharmaceutical Co., Ltd. Seven-week-old male* db/db* mice (BKS.Cg- +Lepr^*db*^/+Lepr^*db*^/Jcl) were purchased from CLEA Japan. These animals were housed under a 12 h : 12 h light/dark cycle (lights on 7:00 a.m.–7:00 p.m.) with controlled room temperature (20°C–26°C) and humidity (35%–75%) and were allowed* ad libitum* access to a diet of CE-2 powder and water. RO5126766 and RO4987655 were administered as a dietary admixture with CE-2.

### 2.4. Animal Experiments

Mice were randomly divided into groups based on body weight (BW) and blood glucose levels by SAS System for Windows, Release 8.02 (SAS Institute Japan, Tokyo, Japan).

For the pharmacological evaluation of RO5126766 and RO4987655, we performed two separate experiments with mice divided into the following groups (*n* = number of animals). In the RO5126766 experiment, each group received 0 mg in 1 kg CE-2 (*n* = 6), 0.86 mg in 1 kg CE-2 (*n* = 5), 1.72 mg in 1 kg CE-2 (*n* = 5), or 3.44 mg in 1 kg CE-2 (*n* = 5). In the RO4987655 experiment, each group received 0 mg in 1 kg CE-2 (*n* = 6), 2 mg in 1 kg CE-2 (*n* = 6), 4 mg in 1 kg CE-2 (*n* = 6), or 8 mg in 1 kg CE-2 (*n* = 6). During the treatment period, food intake (FI) and BW were measured. After 14 days of treatment with the compounds, animals underwent OGTT and then compound admixtures were given for another 3 days. Thereafter, under anesthesia with isoflurane, blood samples were taken and animals were killed, and insulin-responsive tissues were harvested for western blotting.

For the analysis of the mode of action of these compounds, hyperinsulinemic clamp tests were performed after treatment with each of the compounds for 9–11 days in two separate experiments. Mice were divided into the following groups (*n* = number of animals): control (*n* = 10), RO5126766 (2 mg in 1 kg CE-2, *n* = 10), and pioglitazone (200 mg in 1 kg CE-2, *n* = 5); and control (*n* = 10), RO4987655 (8 mg in  1 kg CE-2, *n* = 9), and pioglitazone (200 mg in 1 kg CE-2, *n* = 10).

For the evaluation of the effect of MEK1 knockdown, we administered shRNA of* Mek1* intravenously to* db/db* mice; after 3 days, blood glucose, BW, and FI were measured, the animals were killed under anesthesia with isoflurane, and then the liver was taken for western blotting and qRT-PCR.

### 2.5. Oral Glucose Tolerance Test (OGTT)

After administration of RO5126766 and RO4987655 as a dietary admixture for 14 days, mice were fasted overnight. Next morning, blood glucose was measured at 30, 60, 120, and 240 min after oral administration of glucose (2 g·kg^−1^ of 20% glucose solution) using Accu-Chek Aviva (Roche Diagnostics, Tokyo, Japan).

### 2.6. Hyperinsulinemic-Euglycemic Clamp Test

The hyperinsulinemic-euglycemic clamp test was performed as previously described [1] with slight modifications. RO5126766 and RO4987655 were administered as a dietary admixture to* db/db *mice as described above. On day 7 or 8, two jugular vein cannulae were inserted under anesthesia (sodium pentobarbital, 60 mg·kg^−1^, additional as appropriate) and analgesia (bupivacaine hydrochloride, 0.5%) and then passed subcutaneously to the back. Then the cannulae were connected to swivels through a tether, and the swivels were fixed to a metal rack. Heparin solution (50 U·mL^−1^ at the rate of 12 *µ*L·h^−1^) was continuously infused with a daily flush (50 *µ*L·head^−1^) until the day of the clamp test. After a 2- to 4-day recovery period with continuing feeding of the dietary admixture, on day 10 or 11, the hyperinsulinemic-euglycemic clamp test was conducted. In the morning, diet was removed and [U-^13^C]glucose infusion (0.5 mg·kg^−1^·min^−1^) was started. After 2 h, blood was collected for the measurement of basal endogenous glucose production (EGP); thereafter, insulin (25 IU·kg^−1^·min^−1^) infusion was started. Blood glucose level was monitored by Accu-Chek Aviva at 10 min intervals. When blood glucose reached nearly 110 mg·dL^−1^, glucose (10%) infusion was initiated to maintain blood glucose at 110 mg·dL^−1^. Glucose infusion rate (GIR) was calculated from the following calculation formula, which is a slightly modified version of a previously described formula [29],

(1) I2=I1Ts−TdTs+I0TdTs+VGD−GMkpTs+VGP−GMkdTs,

where *I*_2_, *I*_1_, and *I*_0_ are the GIR (mg·kg^−1^·min^−1^), *T*_*s*_ is the sampling interval (= 10 min), *T*_*d*_ is the delay time taken to adjust the glucose infusion pump after measuring blood glucose (= 2 min), GD is the target blood glucose level (= 110 mg·dL^−1^), GM is the current blood glucose level, GP is the blood glucose just 10 min before, *V* is the initial volume of distribution of glucose in *db*/*db* mice (= 16% of BW), and *k*_*p*_ and *k*_*d*_ are correction coefficients (1 or 2).

When GIR and blood glucose reached a steady state, the mean GIR for the previous 60 min (150–210 min in most cases) was calculated and blood was collected to measure the clamp state EGP and insulin level. All mice were euthanized by exsanguination under anesthesia (sodium pentobarbital, 60 mg·kg^−1^, additional as appropriate) and analgesia (bupivacaine hydrochloride, 0.5%) at the end of the clamp period. Plasma insulin was measured by insulin ELISA kit according to the instruction manual. Plasma [U-^13^C]glucose concentrations, together with that of an internal standard (fructose), were determined with an HPLC-MS/MS system (Shimadzu 20A, Shimadzu, Kyoto, Japan; API 4000, AB Sciex, Framingham, MA, USA) with an improved procedure to increase the sensitivity by Cs^+^ attachment to the sugars [1, 30]. The rate of EGP was calculated according to the following equation, as previously described [1, 31]:

(2) $$\begin{array}{c} \text{EGP}=f\left(\;\frac{\text{I}{\text{E}}_{\text{infusate}}}{\text{I}{\text{E}}_{\text{plasma}}}-1\;\right), \end{array}$$

where EGP is the rate of endogenous glucose production, *f* is the infusion rate of [U-^13^C]glucose (0.5 mg·kg^−1^·min^−1^), IE_infusate_ is the isotopic enrichment of [U-^13^C]glucose in infusate, and IE_plasma_ is the isotopic enrichment (%) of [U-^13^C]glucose in plasma (= plasma [U-^13^C]glucose concentration/total plasma glucose concentration × 100).

### 2.7. Adenovirus-Mediated shRNA* Mek1* Treatment

The adenovirus was prepared by the BLOCK-iT Adenoviral RNAi Expression System (Life Technology, Carlsbad, CA, USA) according to the instruction manual. The following sequences were used: shRNA* Mek1* sequence* Map2k1*: 5′-GGCAGCTAATTGACTCTATGGCGAACCATAGAGTCAATTAGCTGCC-3′, and scramble sequence: 5′-GGACTCGGGCCACCGGGTACGAATACCCGGTGGCCCGAGTCC-3′.

A Fast-Trap Virus Purification/Concentration Kit (Millipore, Billerica, MA, USA) was used for purification and concentration of the adenovirus according to the instruction manual. Titer was determined using an Adeno-X Rapid Titer Kit (Clontech, Mountain View, CA, USA) according to the instruction manual, and infectious units (ifu) were calculated. The adenovirus was diluted to 5 × 10^10^ ifu·10 mL^−1^ with 20 mmol·L^−1^ Tris-HCl, pH 8.0, containing 2% glycerol, and 200 mmol·L^−1^ NaCl. The adenovirus was injected at a volume of 10 mL·kg^−1^ through the tail vein into* db/db* mice fed a normal diet. Blood glucose, BW, and FI were measured 3 days after administration of the adenovirus under unanesthetized conditions. All mice were euthanized by exsanguination under anesthesia (isoflurane) at the end of the experimental period.

### 2.8. Antibodies

Phospho-p44/42 MAPK (ERK1/2) (Thr^202^/Tyr^204^, E10) antibody, p44/42 MAPK (ERK1/2) antibody, MEK1 antibody, MEK2 antibody, and horseradish peroxidase-conjugated secondary anti-rabbit and anti-mouse antibodies were purchased from Cell Signaling Technology (Beverly, MA, USA). Anti-tubulin alpha antibody, as a reference antibody, was purchased from Serotec (Oxford, UK). Horseradish peroxidase-conjugated secondary anti-rat antibody was purchased from Zymed (Carlsbad, CA, USA).

### 2.9. Western Blot Analysis

Western blot analysis was performed by the method previously reported [32, 33] with slight modifications. Tissue lysates were prepared using RIPA buffer in combination with protease inhibitors, phosphatase inhibitors, 1 mmol·L^−1^ EDTA, and 2 mmol·L^−1^ phenylmethylsulfonyl fluoride. After centrifugation (20,000 ×g for 10 min, 4°C), supernatant was collected and protein concentration was measured using bovine serum albumin as a standard. Aliquots of protein from the supernatant were dissolved in Laemmli sample buffer [34] containing 1 mmol·L^−1^ DTT and heated to 95°C for 5 min. Equal amounts of protein from the sample buffer were loaded in each lane, resolved in SDS-PAGE, and transferred to PVDF membranes, which were incubated overnight at 4°C with specific primary antibodies. After incubation with horseradish peroxidase-conjugated secondary antibodies, membranes were incubated with chemiluminescent substrate and were detected using a Fujifilm LAS-4000 apparatus (Fujifilm Life Science, Tokyo, Japan). Some membranes were subsequently reprobed with the indicated antibody as a loading control. Quantifications were realized using MultiGauge software version 3.2.0.0 (Fujifilm Life Science).

### 2.10. Quantitative Real-Time Polymerase Chain Reaction (qRT-PCR)

Tissue was lysed in RLT buffer (Qiagen, Limburg, Netherlands), and RNA was extracted from RLT buffer by using an RNeasy Mini kit (Qiagen) following the manufacturer's instructions. RNA yield and quality were determined by using a NanoDrop (NanoDrop Technologies, Wilmington, DE, USA). Real-time PCR reactions were performed with a 7900HT Fast Sequence Detection System (Life Technologies Japan, Tokyo, Japan). Relative mRNA levels were calculated with a delta-delta-Ct method normalized to 18S rRNA levels as an internal control using the following primers: TaqMan Gene Expression Assay for Mouse* Map2k1* (Applied Biosystems, Carlsbad, CA, USA; Assay ID: Mm00435940_m1) and Predeveloped TaqMan Assay Reagents 18S rRNA (20x) (Applied Biosystems, Catalog Number 4319413E).

### 2.11. Statistical Analysis

Data are expressed as mean ± SEM. Statistical analysis was performed with SAS System for Windows, Release 8.02 (SAS Institute Japan). Statistical significance was determined by the parametric Dunnett's multiple comparison or Welch's test. *p* values less than 0.05 were considered to be statistically significant.

## 3. Results

### 3.1. Effects of RO5126766 and RO4987655 on Blood Glucose in* db/db* Mice

**Figure 2:**
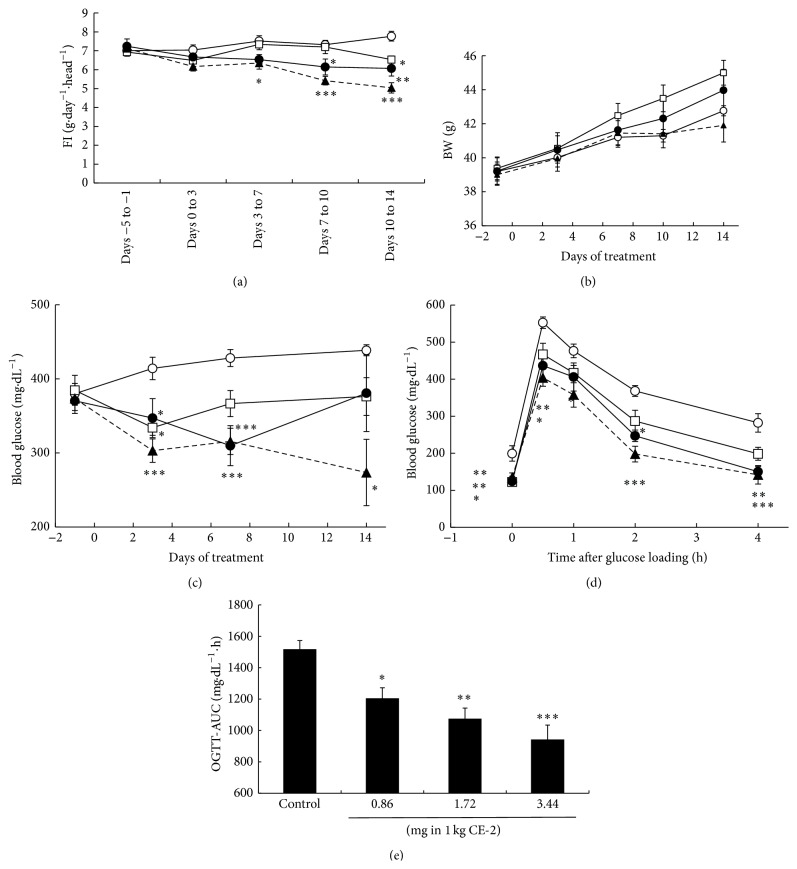
Effects of RO5126766 on (a) food intake (FI), (b) body weight (BW), (c) blood glucose, (d) glucose excursion, and (e) AUC during the oral glucose tolerance test (OGTT) in* db/db* mice, with RO5126766 administered for 14 days as a dietary admixture with dosages as indicated: open circle, 0 mg in 1 kg CE-2; open square, 0.86 mg in 1 kg CE-2; closed circle, 1.72 mg in 1 kg CE-2; and closed triangle, 3.44 mg in 1 kg CE-2. Data are expressed as mean ± SEM, *n* = 6 (control group, 0 mg in 1 kg CE-2) or 5. ^*∗*^ *p* < 0.05, ^*∗∗*^ *p* < 0.01, and ^*∗∗∗*^ *p* < 0.001 versus control at the same measurement time by parametric Dunnett's multiple comparison test.

**Figure 3:**
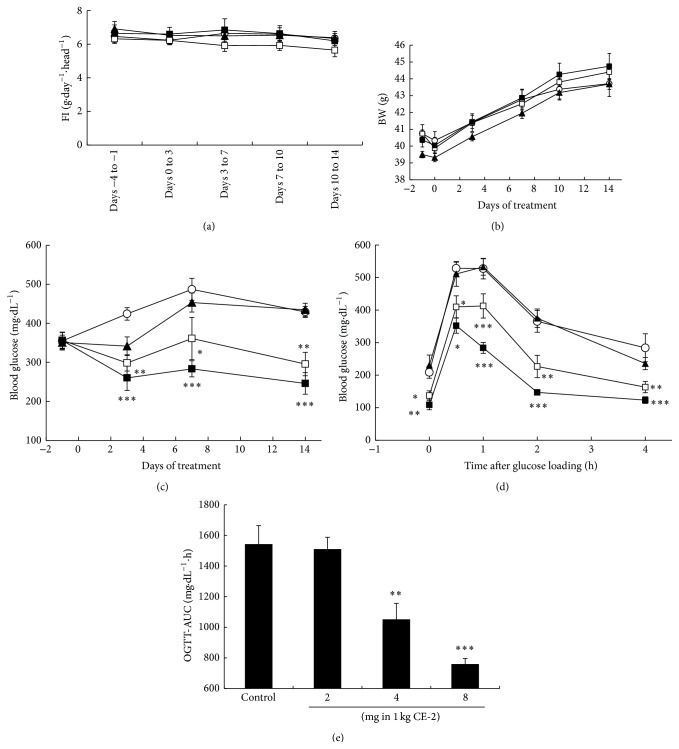
Effects of RO4987655 on (a) food intake (FI), (b) body weight (BW), (c) blood glucose, (d) glucose excursion, and (e) AUC during the oral glucose tolerance test (OGTT) in* db/db* mice, with RO4987655 administered for 14 days as a dietary admixture with dosages as indicated: open circle, 0 mg in 1 kg CE-2; closed triangle, 2 mg in 1 kg CE-2; open rectangle, 4 mg in 1 kg CE-2; and closed rectangle, 8 mg in 1 kg CE-2. Data are expressed as mean ± SEM, *n* = 6. ^*∗*^ *p* < 0.05, ^*∗∗*^ *p* < 0.01, and ^*∗∗∗*^ *p* < 0.001 versus control (0 mg in 1 kg CE-2) at the same measurement time by parametric Dunnett's multiple comparison test.

Three different doses of RO5126766 or RO4987655 were administrated as dietary admixtures to* db/db* mice for 14 days. Mean dosages of RO5126766 and RO4987655 during the administration period were 0.14, 0.27, and 0.49 mg·kg^−1^·day^−1^ and 0.31, 0.57, and 1.25 mg·kg^−1^·day^−1^, respectively (data not shown). At the mid and high dosages, these compounds inhibited around 20% or more of pERK1/2 in peripheral blood mononuclear cells (PBMCs) at the end of the experimental period (Supplemental Figure  S1 in Supplementary Material available online at http://dx.doi.org/10.1155/2016/8264830). Blood glucose decreased in a dose-dependent manner for both RO5126766 (Figure 2(c)) and RO4987655 (Figure 3(c)). During treatment with RO4987655, no effects on food intake (FI, Figure 3(a)) or BW gain (Figure 3(b)) were observed; however, during treatment with RO5126766, an approximately 30% reduction in mean FI was observed at the highest dose (Figure 2(a)) while there was no change in BW (Figure 2(b)).

After 14-day administration, mice were fasted overnight and OGTT was performed. The OGTT showed that, for both compounds, fasting blood glucose (FBG) decreased and glucose tolerance improved (Figures 2(d) and 3(d)) and the area under the curve (AUC) during OGTT was reduced (Figures 2(e) and 3(e)) in a dose-dependent manner.

Since mice treated with the highest dose of RO5126766 (3.44 mg in 1 kg CE-2) showed a 30% reduction in FI (Figure 2(a)), we used untreated age-matched* db/db* mice to confirm that a 30% restriction of FI in itself did not lower the blood glucose level or AUC during OGTT to the same level as observed in RO5126766-treated mice (Figures 2(c)–2(e), Supplemental Figures S4(c)–S4(e)).

During the OGTT, plasma insulin levels in both RO5126766- and RO4987655-treated* db/db* mice were comparable to that of control mice (Supplemental Figure S5). There were no obvious changes in laboratory test values in plasma from* db/db* mice after 17 days of treatment with either compound (Supplemental Tables S2 and S3).

### 3.2. Effects of RO5126766 and RO4987655 on pERK1/2 in Insulin-Responsive Tissues from* db/db* Mice

**Figure 4:**
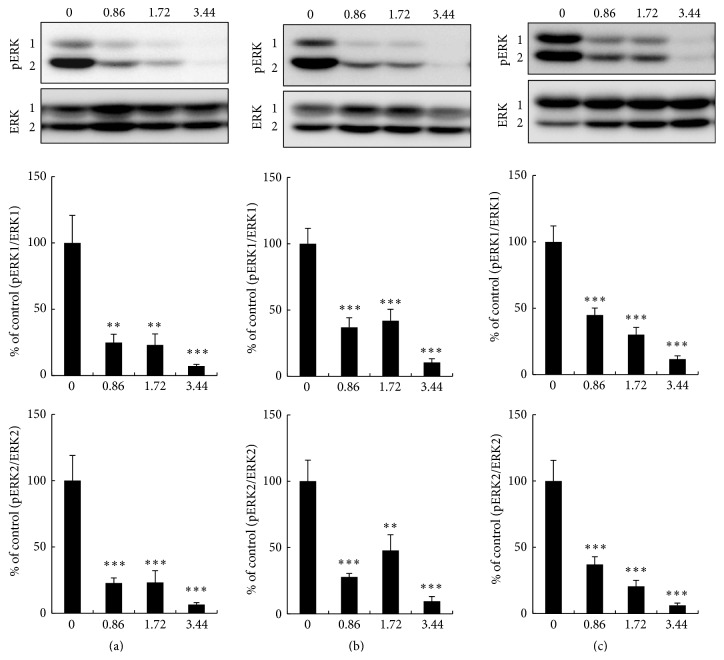
Effects of RO5126766 on pERK1/2 in (a) liver, (b) gastrocnemius muscle, and (c) epididymal adipose tissue from* db/db* mice. RO5126766 was administered as a dietary admixture at the indicated dosages (mg in 1 kg CE-2; numbers on the *x*-axis) for 17 days. Representative blots from each type of tissue are shown at the top of the figures. Data are expressed as mean ± SEM, *n* = 5 or 6 (control group, 0 mg in 1 kg CE-2). ^*∗∗*^ *p* < 0.01, ^*∗∗∗*^ *p* < 0.001 versus control by parametric Dunnett's multiple comparison test.

**Figure 5:**
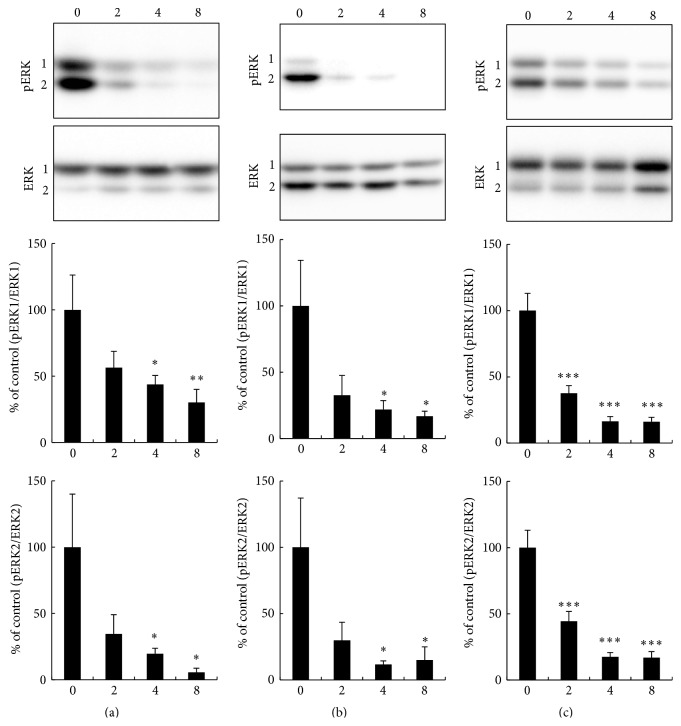
Effects of RO4987655 on pERK1/2 in (a) liver, (b) soleus muscle, and (c) epididymal adipose tissue from* db/db* mice. RO4987655 was administered as a dietary admixture at the indicated dosages (mg in 1 kg CE-2; numbers on the *x*-axis) for 17 days. Representative blots from each type of tissue are shown at the top of the figures. Data are expressed as mean ± SEM, *n* = 6. ^*∗*^ *p* < 0.05, ^*∗∗*^ *p* < 0.01, and ^*∗∗∗*^ *p* < 0.001 versus control group (0 mg in 1 kg CE-2) by parametric Dunnett's multiple comparison test.

To make sure that the MEK inhibition occurred in insulin-responsive tissues as well as in PBMC, pERK1/2 was determined by western blotting in insulin-responsive tissues from* db/db* mice to which RO5126766 (Figure 4) or RO4987655 (Figure 5) had been administered for 17 days. Phosphorylation of ERK1/2 was dose-dependently inhibited in the liver (Figures 4(a) and 5(a)), gastrocnemius muscle (Figure 4(b)) and soleus muscle (Figure 5(b)), and epididymal adipose tissue (EAT, Figures 4(c) and 5(c)) by both RO5126766 and RO4987655. RO5126766 administered at 0.86 mg in 1 kg CE-2 or RO498655 administered at 4 mg in 1 kg CE-2 resulted in much higher inhibition of pERK1/2 in all tested tissues than was observed in PBMCs (Supplemental Figure S1).

### 3.3. Hyperinsulinemic-Euglycemic Glucose Clamp Test

To explore the mode of action through which MEK inhibition lowers glucose, a dosage of either 2 mg of RO5126766 or 8 mg of RO4987655 in 1 kg CE-2 was administered to* db/db* mice for 9–11 days, and 200 mg of pioglitazone in 1 kg CE-2 was administered to a separate group of mice as a reference. A hyperinsulinemic-euglycemic glucose clamp test was conducted after 4 h fasting on the day of the clamp test. No decrease in FI or BW was observed during the treatment (data not shown).

FBG was significantly decreased by administration of RO5126766, and GIR was significantly increased to 26.8 ± 2.7 mg·kg^−1^·min^−1^ compared to that of control (9.5 ± 2.1 mg·kg^−1^·min^−1^) (Table 1), suggesting that IR was improved. EGP from mice treated with RO5126766 was significantly decreased from 16.8 ± 3.7 mg·kg^−1^·min^−1^ in control mice to 6.8 ± 2.3 mg·kg^−1^·min^−1^ (Table 1), suggesting improvement of IR in the liver. Decreased EGP (16.8 − 6.8 = 10.0 mg·kg^−1^·min^−1^) by RO5126766 administration accounted for around 58% of the increased GIR (26.8 − 9.5 = 17.3 mg·kg^−1^·min^−1^). Moreover, plasma insulin level at the end of the clamp test was comparable between the three groups (control, 43.7 ± 3.5 ng·mL^−1^; RO5126766, 50.4 ± 6. 4 ng·mL^−1^; and pioglitazone, 41.6 ± 8.8 ng·mL^−1^; Table 1), which supports the improvement of IR by RO5126766.

Similar results were obtained when RO4987655 was administered to mice. FBG was significantly decreased and GIR was significantly increased to 28.2 ± 3.7 mg·kg^−1^·min^−1^ compared to that of control (19.0 ± 1.3 mg·kg^−1^·min^−1^) (Table 2), suggesting that RO4987655 improved IR as well. EGP was not statistically significantly different (*p* = 0.1779) but tended to decrease, from 11.9 ± 1.8 mg·kg^−1^·min^−1^ in control mice to 6.5 ± 1.9 mg·kg^−1^·min^−1^ (Table 2), suggesting that the IR in the liver was improved by RO4987655. Decreased EGP (11.9 − 6.5 = 5.4 mg·kg^−1^·min^−1^) by RO4987655 administration accounted for around 59% of the increased GIR (28.2 − 19.0 = 9.2 mg·kg^−1^·min^−1^). Plasma insulin level at the end of the clamp test was comparable between the control group (40.1 ± 2.6 ng·mL^−1^) and the RO4987655-treated group (28.1 ± 4.6 ng·mL^−1^) (Table 2).

Approximately 60% of the increase in GIR following the administration of either RO4987655 or RO5126766 is explained by the suppression of EGP, which appears to be mainly caused by MEK1/2 inhibition improving IR in the liver.

Similarly, decreased FBG and increased GIR were observed in mice treated with pioglitazone (average dose, 30 mg·kg^−1^·day^−1^). The percentages of decreased EGP that accounted for the increased GIR were 88% for RO5126766 (Table 1) and 57% for RO4987655, respectively (Table 2).

### 3.4. Suppression of MEK1 Protein in the Liver by Adenovirus-Mediated shRNA of* Mek1 (Map2k1)* Expression and Its Effect on Blood Glucose

Because the hyperinsulinemic-euglycemic glucose clamp test revealed that the tissue in which IR was most improved by MEK inhibitions would be the liver, we hypothesized that suppression of MEK1 protein expression in the liver would lead to decreased blood glucose in* db/db* mice. We tested the hypothesis by using adenovirus-mediated shRNA expression of* Mek1* in* db/db* mice.

**Figure 6:**
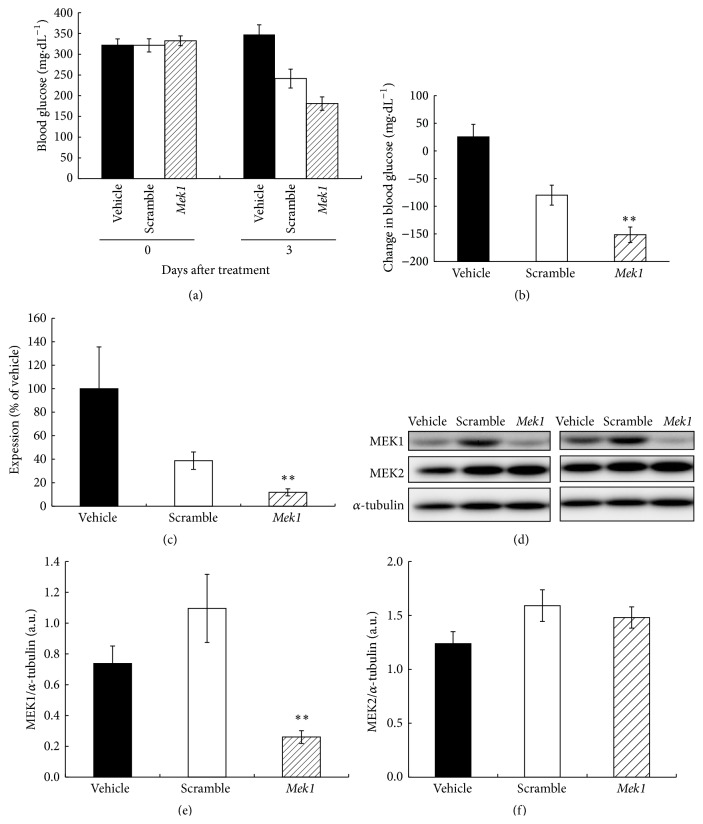
Effects of* Mek1 (Map2k1)* silencing by adenovirus-mediated shRNA expression of* Mek1* in* db/db* mice on (a, b) blood glucose, (c)* Mek1* mRNA expression level in the liver, (d) representative blots of MEK1 and MEK2 protein in the liver, and (e, f) MEK1 and MEK2 protein expression level in the liver. Data are expressed as mean ± SEM, *n* = 6. ^*∗∗*^ *p* < 0.01 versus scramble by Welch's test. The lanes were run on the same gel but were noncontiguous. Black bars, vehicle-treated group; white bars, scramble-treated group; hatched bars,* Mek1*-silenced group.

Three days after administration of an adenovirus which expressed shRNA of* Mek1* (5 × 10^10^ ifu·kg^−1^), blood glucose tended to be lower than that in the group treated with scrambled shRNA (scramble-treated group) (Figure 6(a)), and the change in blood glucose was significantly lower than that of the scramble-treated group (Figure 6(b)).* Mek1* mRNA level determined by qRT-PCR and MEK1 protein expression determined by western blotting in the liver were decreased to 20–30% of those of scramble-treated mice (Figures 6(c)–6(e)). A compensatory increase in MEK2 protein expression was not observed (Figures 6(d) and 6(f)). There were no effects on FI and BW gain by the adenovirus-mediated shRNA expression (data not shown).

## 4. Discussion

We showed here that RO5126766 and RO4987655 exerted antidiabetic effects in* db/db* mice. These antidiabetic effects were not due to “off-target” effects of these compounds because (i) these compounds are structurally different (Figure 1), (ii) RO5126766 is specific to Raf/MEK and 10 *µ*mol·L^−1^ RO5126766 did not inhibit any of the 256 other kinases in the Ambit KINOME scan panel [24, 26], (iii) RO4987655 is specific to MEK1/2 and 10 *µ*mol·L^−1^ RO4987655 did not inhibit any of the 400 other kinases [28], and (iv) there were no toxic signs in* db/db* mice treated with these compounds for 17 days (Supplemental Tables S2 and S3). These results are further supported by similar results obtained from KK-Ay mice (Supplemental Figure S2), which is another T2D model animal that has a different genetic background from* db/db* mice but which shows similar phenotypes, such as obesity, hyperglycemia, and hyperinsulinemia [35, 36]. Taken together, these results strongly suggest that MEK inhibition could lower blood glucose in T2D.

The two MEK inhibitors used in this experiment did not induce much BW gain in either* db/db* or KK-Ay mice (Figures 2 and 3, Supplemental Figures S2 and S3). In addition, they did not show any effect on the expression of several adipogenic genes that are downstream of the PPAR*γ* pathway (Supplemental Figure S6) nor did they have any effect on insulinotropic action (Supplemental Figure S5). We also did not observe any direct insulinotropic action on INS-1E cells by these compounds (data not shown).

The results from the hyperinsulinemic-euglycemic glucose clamp test in the animals treated with the MEK inhibitors strongly suggested whole-body improvement of IR similar to that seen in mice treated with pioglitazone (Tables 1 and 2). Furthermore, with both compounds, about 60% of the increased GIR was accounted for by decreased EGP (Tables 1 and 2), suggesting that the liver would be the tissue most responsive to whole-body improvement of IR by MEK inhibition. However, in compound-treated mice, dose-dependent inhibition of pERK1/2 was also observed in other insulin-responsive tissues, such as soleus muscle, and EAT (Figures 4 and 5), which suggests that although the main responsive tissue is liver, peripheral tissues, that is, skeletal muscle and adipose tissue, would contribute to amelioration of the remaining part of whole-body IR* in vivo*. We observed a tendency for Akt Ser^473^ phosphorylation to increase in soleus muscle and EAT, as well as in the liver from* db/db* mice after administration of both compounds (data not shown). The underlying molecular mechanisms need to be analyzed in further detail to understand the exact relationships between MEK inhibition and amelioration of IR, which will lead to deeper insights into alternative therapeutic strategies for treating IR or T2D.

Because the improvement of IR was achieved mainly in the liver, we confirmed whether suppressing MEK1 protein expression in the liver of* db/db* mice would lead to decreased blood glucose. We found that adenovirus-mediated shRNA expression of* Mek1* could suppress the MEK1 protein expression only in the liver (not in skeletal muscle, data not shown). Administration of adenovirus-mediated shRNA of* Mek1 *reduced blood glucose (Figures 6(a) and 6(b)) accompanied by lower levels of* Mek1* mRNA and protein expression in the liver than were observed in the scramble-treated group (Figures 6(c)–6(e)). These results strongly supported our hypothesis that lowering MEK1 activity in the liver would lead to decreased blood glucose. However, in our western blot analysis, phosphorylation of ERK1/2 in the liver was not decreased by the administration of shRNA of* Mek1* (data not shown). This may be due to phosphorylation by intact MEK2 or by the remaining part of MEK1 activity, because MEK1 protein was not completely inhibited (Figures 6(d) and 6(e)). The relatively low degree of decrease in blood glucose levels that was achieved by* Mek1 *silencing as compared to the decrease in blood glucose achieved by MEK inhibitors may be because the inhibitory effect of a compound administered systemically was exerted not only in the liver but also in skeletal muscle and EAT, as we have suggested above. In the group treated with scrambled shRNA, the mRNA level of* Mek1* in the liver decreased to less than half that of the vehicle-treated group (Figure 6(c)); however, MEK1 protein expression level in the liver did not decrease, as shown in Figures 6(d) and 6(e). We do not know the exact mechanism through which the scrambled shRNA decreased blood glucose and* Mek1 *mRNA in this particular experiment* in vivo*. One possible explanation is that a more significant reduction in* Mek1* mRNA expression is necessary for a decrease in MEK1 protein; another possible explanation is that the decrease in blood glucose by an uncertain mechanism of scrambled shRNA may lead to a decrease in the level of* Mek1* mRNA expression. Whatever the case may be, the fact that there was no reduction in MEK1 protein by the scrambled shRNA suggests that the effect of* Mek1* mRNA reduction on blood glucose can be considered to be minimal.

In the present study, we evaluated MEK inhibition as an antidiabetic drug target using a T2D animal model, the* db/db* mouse. Using pharmacological intervention with two structurally different MEK inhibitors (RO5126766 and RO4987655) and RNA interference intervention with adenovirus-mediated* Mek1* shRNA expression, we confirmed that inhibition of MEK activity and suppression of MEK1 protein expression both lowered blood glucose through the amelioration of IR.

## 5. Conclusions

From the current study, we conclude that MEK could be a potential therapeutic target for T2D treatment. The therapeutic potential of MEK inhibition needs to be examined in clinical trials with T2D patients, and further study is needed to explore the exact mechanisms through which inhibiting the MEK1 signaling pathway improves IR, especially in the liver, in order to avoid undesirable adverse events by MEK inhibition [37, 38].

## Supplementary Material

The Supplementary Material contains 3 supplemental tables and 6 figures showing additional data on the basic characteristics of RO5126766 and RO4987655 in Supplemental Table S1 and the following *in vivo* experiment results: From the first pharmacological evaluation of RO5126766 and RO498765 in *db/db* mice, Supplemental Tables S2 and S3 show the background blood chemical values after 17 days of treatment, and Supplemental Figure S1 shows the inhibition of ERK1/2 phosphorylation in peripheral blood mononuclear cells after 17 days of treatment. Supplemental Figure S2 shows antidiabetic effects of RO5126766 and RO4987655 in KK-Ay mice, and Supplemental Figure S3 shows changes in body weight induced by the treatment of pioglitazone in both *db/db* and KK-Ay mice. Additionally, Supplemental Figure S4 shows the effect of food restriction (70%) on blood glucose and glucose excursion during an oral glucose tolerance test (OGTT) in *db/db* mice. Finally, Supplemental Figure S5 shows the effects of RO5126766, RO4987655, and pioglitazone on the plasma insulin level during the OGTT, and Supplemental Figure S6 shows the adipogenic gene expression levels in epididymal adipose tissue in *db/db* mice.

## Figures and Tables

**Table 1:** Blood glucose level, glucose infusion rate (GIR), and endogenous glucose production (EGP) of RO5126766-treated *db/db* mice in the hyperinsulinemic-euglycemic clamp test.

	Control	RO5126766	Pioglitazone
		(2 mg in 1 kg CE-2)	(200 mg in 1 kg CE-2)
*n*	10	10	5
Blood glucose (mg·dL^−1^) (4 h fasted)	272.9 ± 30.6	141.2 ± 12.9^*∗∗∗*^	104.6 ± 12.4^*∗∗∗*^
Blood glucose (mg·dL^−1^)^¶^	121.3 ± 8.7	109.5 ± 2.7	107.9 ± 3.0
GIR (mg·kg^−1^·min^−1^)^¶^	9.5 ± 2.1	26.8 ± 2.7^*∗∗∗*^	29.7 ± 5.7^*∗∗∗*^
EGP (mg·kg^−1^·min^−1^)	16.8 ± 3.7	6.8 ± 2.3^*∗*^	−0.9 ± 3.0^*∗∗*^
Plasma insulin (ng·mL^−1^)	43.7 ± 3.5	50.4 ± 6.4^a^	41.6 ± 8.8

Data are expressed as mean ± SEM.

^¶^Mean values of last 60 min on clamp.

^*∗*^ *p* < 0.05, ^*∗∗*^ *p* < 0.01, and ^*∗∗∗*^ *p* < 0.001 versus control by parametric Dunnett's multiple comparison test.

^a^ *n* = 9.

**Table 2:** Blood glucose level, glucose infusion rate (GIR), and endogenous glucose production (EGP) of RO4987655-treated *db/db* mice in the hyperinsulinemic-euglycemic clamp test.

	Control	RO4987655	Pioglitazone
		(8 mg in 1 kg in CE-2)	(200 mg in 1 kg CE-2)
*n*	10	9	10
Blood glucose (mg·dL^−1^) (4 h fasted)	228.4 ± 34.7	125.4 ± 11.2^*∗∗*^	142.1 ± 16.0^*∗*^
Blood glucose (mg·dL^−1^)^¶^	103.4 ± 3.3	108.7 ± 1.5	108.1 ± 4.6
GIR (mg·kg^−1^·min^−1^)^¶^	19.0 ± 1.3	28.2 ± 3.7^*∗*^	35.8 ± 2.0^*∗∗∗*^
EGP (mg·kg^−1^·min^−1^)	11.9 ± 1.8	6.5 ± 1.9^§^	2.3 ± 2.6^*∗∗*^
Plasma insulin (ng·mL^−1^)	40.1 ± 2.6^a^	28.1 ± 4.6^b^	ND

Data are expressed as mean ± SEM.

^¶^Mean values of last 60 min on clamp.

^*∗*^ *p* < 0.05, ^*∗∗*^ *p* < 0.01, and ^*∗∗∗*^ *p* < 0.001 versus control by parametric Dunnett's multiple comparison test.

^§^ *p* = 0.1779 versus control by parametric Dunnett's multiple comparison test.

ND, not determined.

^a^ *n* = 5 and ^b^ *n* = 3.

## Abbreviations

Akt: Protein kinase B
AUC: Area under the curve
BW: Body weight
EAT: Epididymal adipose tissue
EGP: Endogenous glucose production
ERK: Extracellular signal-regulated kinase
FBG: Fasting blood glucose
FI: Food intake
GIR: Glucose infusion rate
ifu: Infectious units
IR: Insulin resistance
IRS-1: Insulin receptor substrate-1
MAPK: Mitogen-activated protein kinase
MEK: Mitogen-activated protein kinase kinase
OGTT: Oral glucose tolerance test
PBMC: Peripheral blood mononuclear cells
PD: Pharmacodynamics
pERK1/2: Phosphorylation of ERK1/2
PI 3-K: Phosphoinositide 3-kinase
PPAR: Peroxisome proliferator-activated receptor
qRT-PCR: Quantitative real-time polymerase chain reaction
T2D: Type 2 diabetes.
