# Supplementary material for: Inhibition of MEK1 Signaling Pathway in the Liver Ameliorates Insulin Resistance
Source: J Diabetes Res. 2015 Dec 28;2016:8264830. doi: 10.1155/2016/8264830 (PMC4709921; doi:10.1155/2016/8264830)
Supplement: Supplementary file 1 — The Supplementary Material contains 3 supplemental tables and 6 figures showing additional data on the basic characteristics of RO5126766 and RO4987655 in Supplemental Table S1 and the following in vivo experiment results: From the first pharmacological evaluation of RO5126766 and RO498765 in db/db mice, Supplemental Tables S2 and S3 show the background blood chemical values after 17 days of treatment, and Supplemental Figure S1 shows the inhibition of ERK1/2 phosphorylation in peripheral blood mononuclear cells after 17 days of treatment. Supplemental Figure S2 shows antidiabetic effects of RO5126766 and RO4987655 in KK-Ay mice, and Supplemental Figure S3 shows changes in body weight induced by the treatment of pioglitazone in both db/db and KK-Ay mice. Additionally, Supplemental Figure S4 shows the effect of food restriction (70%) on blood glucose and glucose excursion during an oral glucose tolerance test (OGTT) in db/db mice. Finally, Supplemental Figure S5 shows the effects of RO5126766, RO4987655, and pioglitazone on the plasma insulin level during the OGTT, and Supplemental Figure S6 shows the adipogenic gene expression levels in epididymal adipose tissue in db/db mice. [file 8264830.f1.pdf]

# Supplemental data

# Inhibition of MEK1 signaling pathway in the liver ameliorates insulin resistance

**Short running title:** MEK1 inhibition ameliorates insulin resistance

Atsunori Ueyama[1, 4, *], Nobuhiro Ban[1], Masanori Fukazawa[1], Tohru Hirayama[1], Minako Takeda[1], Tatsuo Yata[2], Hiroyasu Muramatsu[1], Masaki Hoshino[1], Marii Yamamoto[1], Masao Matsuo[1], Yuka Kawashima[1], Tatsuhiko Iwase[3], Takehisa Kitazawa[1], Youichi Kushima[1], Yuichiro Yamada[4], and Yoshiki Kawabe[1]

*[1] Research Division, Chugai Pharmaceutical Co., Ltd., [2]Chugai Research Institute for Medical Science, 1-135 Komakado, Gotemba-city, Shizuoka 412-8513, Japan,*

*[3] Project Planning & Coordination Department, Chugai Pharmaceutical Co., Ltd, 2-1-1 Nihonbashi, Muromachi, Chuo-ku, Tokyo 103-8324, Japan,*

*[4] Department of Endocrinology, Diabetes and Geriatric Medicine, Akita University School of Medicine, 1-1-1 Hondo, Akita-city, Akita 010-8543, Japan*

***Corresponding author**

Atsunori Ueyama
Discovery of Pharmacological Research Department 1,
Chugai Pharmaceutical Co., Ltd.,
1-135 Komakado, Gotemba-city, Shizuoka 412-8513, Japan
TEL: +81-550-87-6738, FAX: +81-550-87-5219, E-mail: ueyamaatn@chugai-pharm.co.jp

**3 supplemental tables, 6 supplemental figures, 1 supplemental reference**

**Supplemental Table S1**

$IC_{50}$ values and serum half-life of RO5126766 and RO4987655

| | | RO5126766 | RO4987655 |
|---|---|---|---|
| Kinase inhibition, *in vitro*, $IC_{50}$ ($\mu mol \cdot L^{-1}$) | Raf1 | 0.056 | >50 |
| | MEK1 | 0.16 | 0.0052 |
| Serum half-life (h) | Mouse | 4.5 | 3.5 |
| | Human | 53–95 | 25 |

**Supplemental Table S2**

Effect of RO5126766 on blood chemical values in *db/db* mice after 17 days of treatment. RO5126766 was administrated as a dietary admixture in the indicated dosages (mg in 1 kg CE-2). Plasma was obtained after exsanguination under anesthesia at the end of the administration period. Data are expressed as mean ± SEM.

| | | AST | ALT | ALP | TC | TG | UN | CRE | GLUC |
|---|---|---|---|---|---|---|---|---|---|
| RO5126766 (mg in 1 kg CE-2) | *n* | (U·L$^{-1}$) | (U·L$^{-1}$) | (U·L$^{-1}$) | (mg·dL$^{-1}$) | (mg·dL$^{-1}$) | (mg·dL$^{-1}$) | (mg·dL$^{-1}$) | (mg·dL$^{-1}$) |
| 0 | 6 | 40 ± 3 | 47 ± 3 | 338 ± 11 | 129 ± 4 | 106 ± 10 | 26.8 ± 1.5 | 0.06 ± 0.01 | 682 ± 68 |
| 0.86 | 5 | 39 ± 1 | 52 ± 3 | 248 ± 17 | 132 ± 8 | 110 ± 11 | 29.6 ± 1.0 | 0.05 ± 0.01 | 669 ± 64 |
| 1.72 | 5 | 51 ± 3 | 62 ± 6 | 259 ± 7 | 140 ± 4 | 99 ± 14 | 28.0 ± 0.6 | 0.05 ± 0.00 | 557 ± 26 |
| 3.44 | 5 | 61 ± 6 | 56 ± 6 | 170 ± 17 | 127 ± 3 | 105 ± 4 | 33.9 ± 2.3 | 0.07 ± 0.01 | 468 ± 66 |

AST: aspartate aminotransferase
ALT: alanine aminotransferase
ALP: alkaline phosphatase
TC: total cholesterol
TG: triglycerides
UN: urea nitrogen
CRE: creatinine
GLUC: glucose

**Supplemental Table S3**

Effect of RO4987655 on blood chemical values in *db/db* mice after 17 days of treatment. RO4987655 was administrated as a dietary admixture in the indicated dosages (mg in 1 kg CE-2). Plasma was obtained after exsanguination under anesthesia at the end of the administration period. Data are expressed as mean ± SEM.

| RO4987655 (mg in 1 kg CE-2) | *n* | AST ($U \cdot L^{-1}$) | ALT ($U \cdot L^{-1}$) | ALP ($U \cdot L^{-1}$) | TC ($mg \cdot dL^{-1}$) | TG ($mg \cdot dL^{-1}$) | UN ($mg \cdot dL^{-1}$) | CRE ($mg \cdot dL^{-1}$) | GLUC ($mg \cdot dL^{-1}$) |
|---|---|---|---|---|---|---|---|---|---|
| 0 | 6 | 54 ± 4 | 68 ± 6 | 317 ± 24 | 126 ± 6 | 119 ± 14 | 24.8 ± 1.3 | 0.07 ± 0.01 | 661 ± 20 |
| 2 | 5 | 68 ± 8 | 93 ± 9 | 272 ± 11 | 127 ± 6 | 124 ± 17 | 30.4 ± 2.0 | 0.08 ± 0.01 | 680 ± 28 |
| 4 | 6 | 73 ± 9 | 92 ± 12 | 228 ± 23 | 132 ± 5 | 94 ± 16 | 29.9 ± 1.4 | 0.08 ± 0.01 | 517 ± 38 |
| 8 | 6 | 70 ± 9 | 98 ± 22 | 189 ± 28 | 124 ± 7 | 73 ± 8 | 33.5 ± 3.2 | 0.08 ± 0.01 | 483 ± 49 |

AST: aspartate aminotransferase
ALT: alanine aminotransferase
ALP: alkaline phosphatase
TC: total cholesterol
TG: triglycerides
UN: urea nitrogen
CRE: creatinine
GLUC: glucose

**Supplemental Figure S1**

Inhibition of pERK1/2 in peripheral blood mononuclear cells (PBMC) from *db/db* mice treated for 17 days with (**a**) RO5126766 or (**b**) RO4987655 as a dietary admixture at the indicated dosage (mg in 1 kg CE-2). Phosphorylation of ERK1/2 was detected by flow cytometry. Data are expressed as mean ± SEM, $n$ = 5–6 performed in duplicate. The number (%) indicated above each column shows percentage inhibition when phorbol 12-myristate 13-acetate (PMA)(−) in PBMC from control mice was set as 0% and PMA(+) was set as 100%. ***Method:*** At the end of the 17-day RO5126766 or RO4987655 administration period, blood was collected and incubated with or without 40 μmol·L$^{-1}$ of PMA (Sigma-Aldrich Corporation, St. Louis, MO, USA) for 10 min at 37°C to activate the MAPK pathway in the blood cells. Following PMA stimulation, blood cells were fixed and red blood cells were lysed by the addition of 1× Lyse/Fix buffer (BD Biosciences, Franklin Lakes, NJ, USA), which allows samples to maintain their *in vivo* MEK/ERK status. After lysis and fixation, supernatant was removed and then Perm Buffer II (BD Biosciences) was added to the resulting cells. After washing, the resulting cells were stained with two antibodies (Alexa Fluor 488-conjugated phospho-p44/42 MAPK antibody [ERK1/2; Thr$^{202}$/Tyr$^{204}$]; Cell Signaling Technology, Beverly, MA, USA) and PE-conjugated hamster anti-mouse CD3e antibody (BD Biosciences). The CD3e antibody was used to identify T-lymphocytes, thus allowing pERK1/2 levels to be analyzed only in the gated CD3-positive lymphocyte population. Phosphorylation of ERK1/2 in PBMCs was assessed by using flow cytometry. The mean fluorescence intensity was measured.

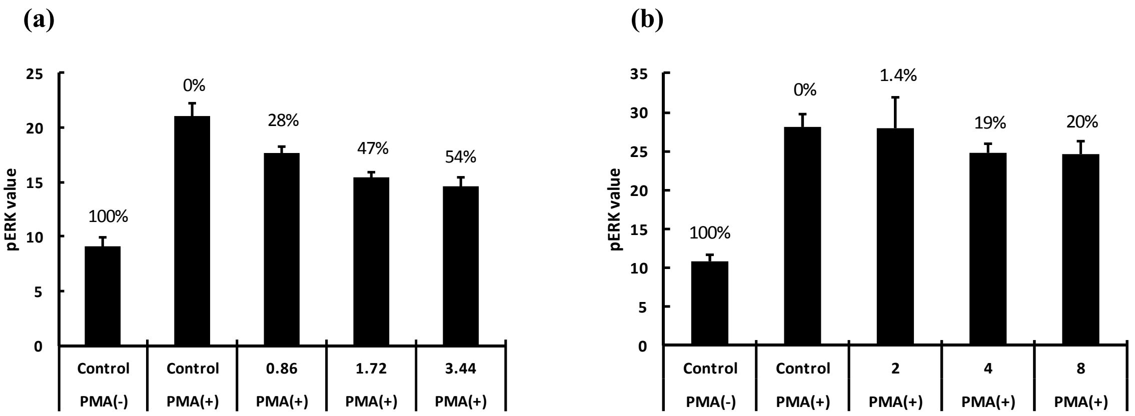

**Supplemental Figure S2**

Effects of RO5126766 (**a, c, e, g, i, k**) and RO4987655 (**b, d, f, h, j, k**) on (**a, b**) blood glucose (BG), (**c, d**) glucose excursion, (**e, f**) AUC during the oral glucose tolerance test (OGTT), (**g, h**) food intake (FI), (**i, j**) body weight (BW), and (**k**) HbA1c in 8-week-old male KK-Ay/Ta Jcl mice (CLEA Japan, Tokyo, Japan). RO5126766 or RO4987655 was administrated for 14 days as a dietary admixture in the dosages indicated (mg in 1 kg CE-2). OGTT (2 $g \cdot kg^{-1}$ of 20% glucose solution) was performed after 14 days of treatment. Hemoglobin A1c (%) was calculated as HbA1c ($g \cdot dL^{-1}$) divided by total Hb ($g \cdot dL^{-1}$). IFCC HbA1c values ($mmol \cdot mol^{-1}$) were converted at http://www.ngsp.org/convert1.asp. Data are expressed as mean ± SEM, $n = 6$. *$p < 0.05$, **$p < 0.01$, ***$p < 0.001$ *vs*. Control by Dunnett's multiple-comparison test.

**(a)**

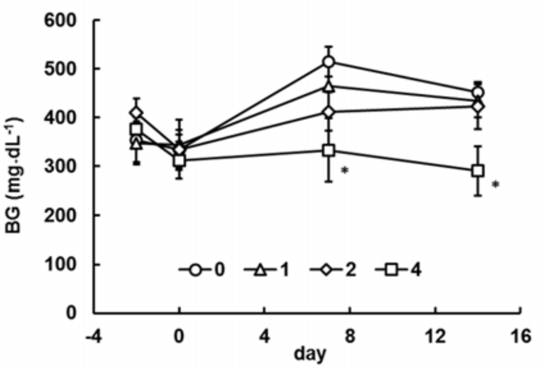


**(b)**

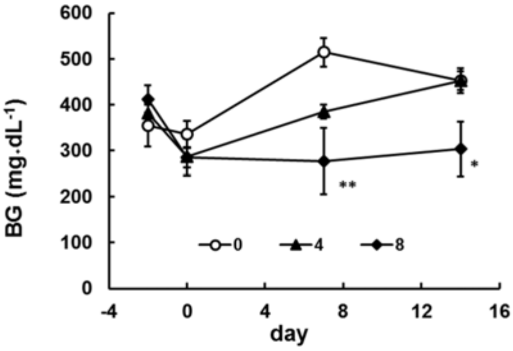


**(c)**

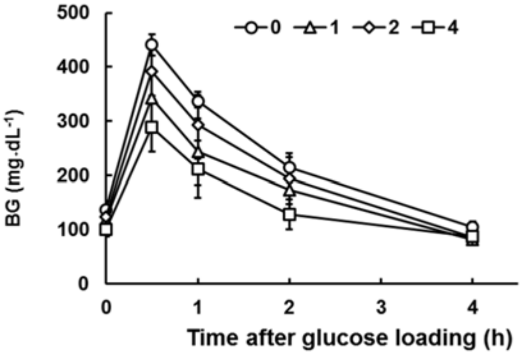


**(d)**

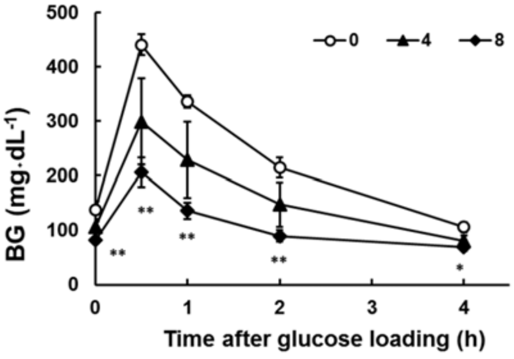

**(e)**

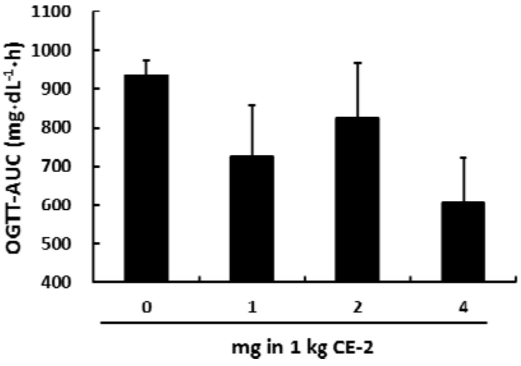
OGTT-AUC (mg·dL⁻¹·h)
1100
1000
900
800
700
600
500
400
0
1
2
4
mg in 1 kg CE-2

**(f)**

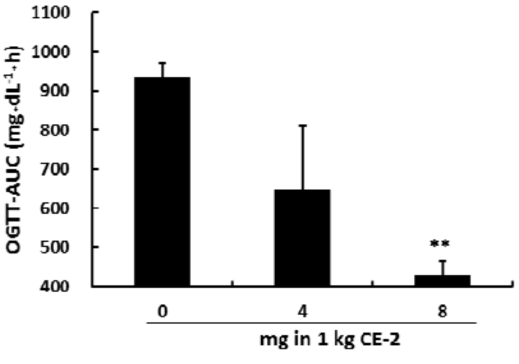
OGTT-AUC (mg·dL⁻¹·h)
1100
1000
900
800
700
600
500
400
0
4
8
mg in 1 kg CE-2

**(g)**

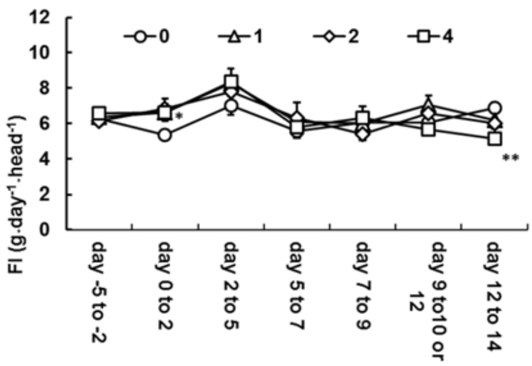
FI (g·day⁻¹·head⁻¹)
0
1
2
4
day -5 to -2
day 0 to 2
day 2 to 5
day 5 to 7
day 7 to 9
day 9 to10 or 12
day 12 to 14

**(h)**

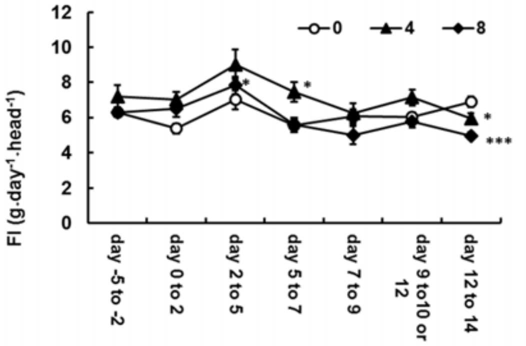
FI (g·day⁻¹·head⁻¹)
0
4
8
day -5 to -2
day 0 to 2
day 2 to 5
day 5 to 7
day 7 to 9
day 9 to10 or 12
day 12 to 14

**(i)**

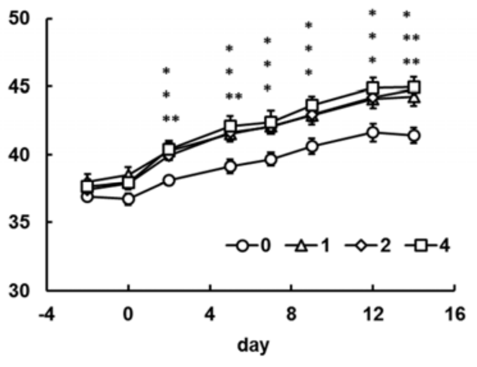
BW (g)
day
0
1
2
4

**(j)**

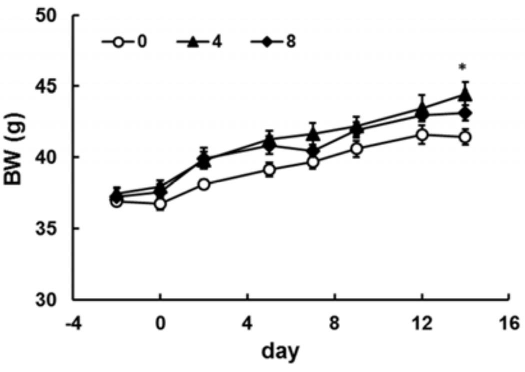
BW (g)
day
0
4
8

**(k)**

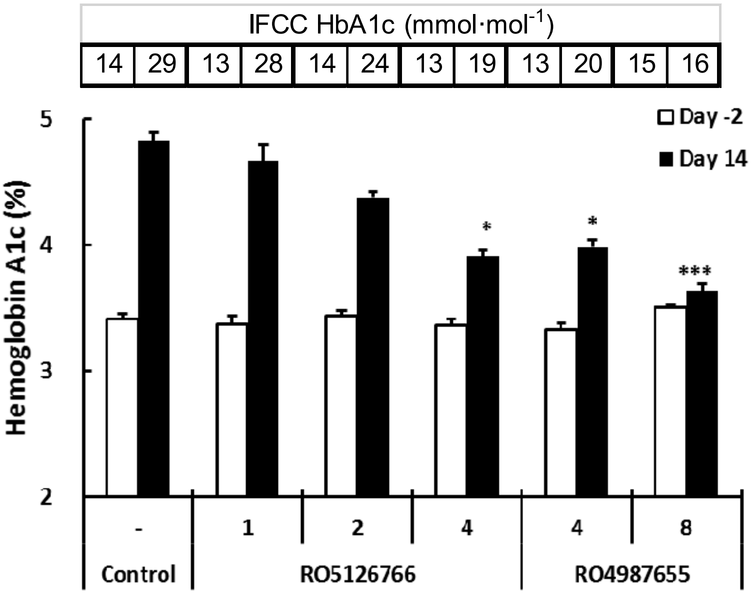
IFCC HbA1c (mmol·mol-1)
14 29 13 28 14 24 13 19 13 20 15 16
Day -2
Day 14
Hemoglobin A1c (%)
5
4
3
2
*
*
***
-
1
2
4
4
8
Control
RO5126766
RO4987655

**Supplemental Figure S3**

Effects of pioglitazone (200 mg in 1 kg CE-2) on (**a, b**) body weight (BW) and (**c**) blood glucose (BG) in **(a)** *db/db* or (**b, c**) KK-Ay mice. Data are expressed as mean ± SEM, $n = 6$. $^{§}p < 0.01$ *vs.* Control (0 mg in 1 kg CE-2) by Dunnett's multiple-comparison test, $^{**}p < 0.01$, $^{***}p < 0.001$ *vs.* Control by Welch's test. Mean dosage of pioglitazone (200 mg in 1 kg CE-2) was about 30 mg·kg$^{-1}$·day$^{-1}$.

**(a)**

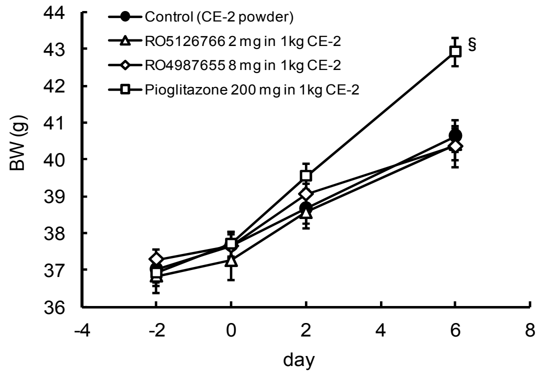


**(b)**

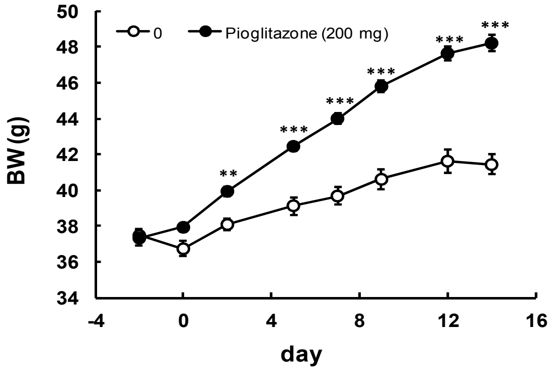


**(c)**

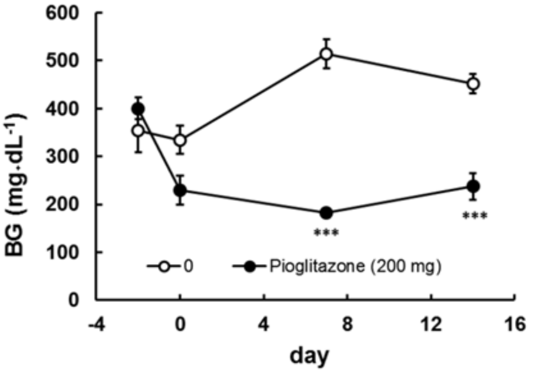

**Supplemental Figure S4**

Effect of food restriction (70%) on (**a**) food intake (FI), (**b**) body weight, (**c**) blood glucose, (**d**) oral glucose tolerance test (OGTT) (2 g·kg$^{-1}$ of 20% glucose solution), and (**e**) AUC of OGTT in *db/db* mice. Seven-week-old *db/db* mice were fed *ad lib* (master, 100%; slave, 70%) using a synchronized pellet pair-feeding apparatus (PairMex, Osaka Micro Systems, Settsu, Japan [1]) for 13 days. Data are expressed as (**a**) mean ± SD or (**b–e**) mean ± SEM, *n* = 4.

**(a)**

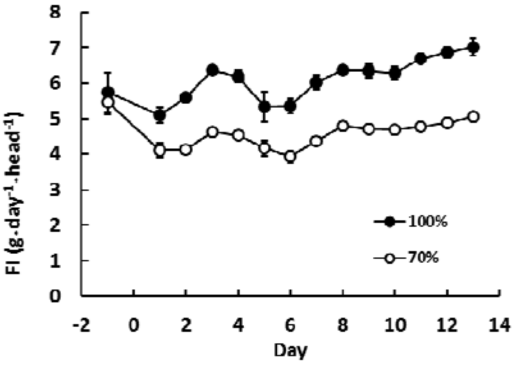


**(b)**

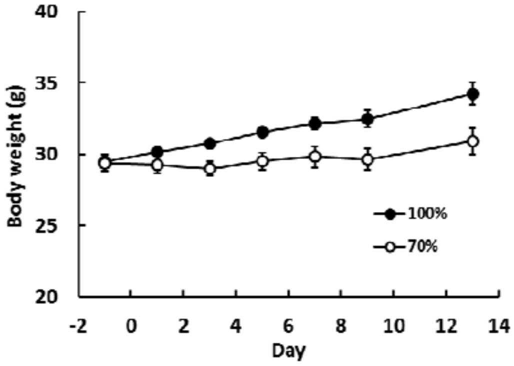


**(c)**

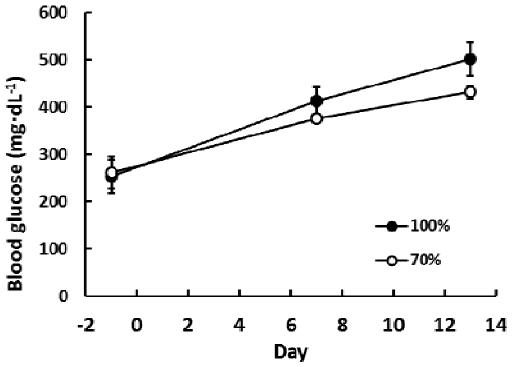


**(d)**

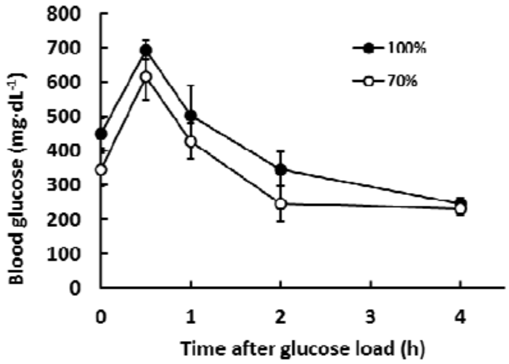


**(e)**

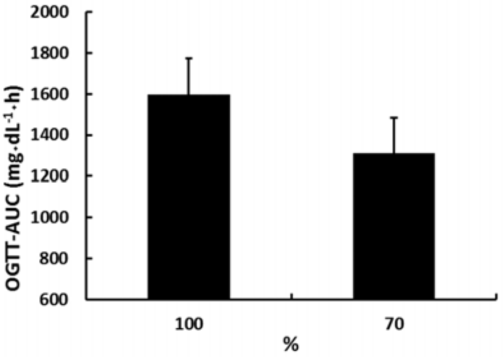

**Supplemental Figure S5**

The effect of RO5126766 and RO4987655 on plasma insulin level during the oral glucose tolerance test in *db/db* mice. After administration of these compounds as a dietary admixture for 14 days, mice were fasted overnight. Next morning, blood glucose was measured, followed by oral administration of glucose (2 g·kg$^{-1}$ of 20% glucose solution). Plasma insulin level was measured by insulin ELISA kit (Morinaga Institute of Biological Science, Inc., Yokohama, Japan). Data are expressed as mean ± SEM, *n* = 8.

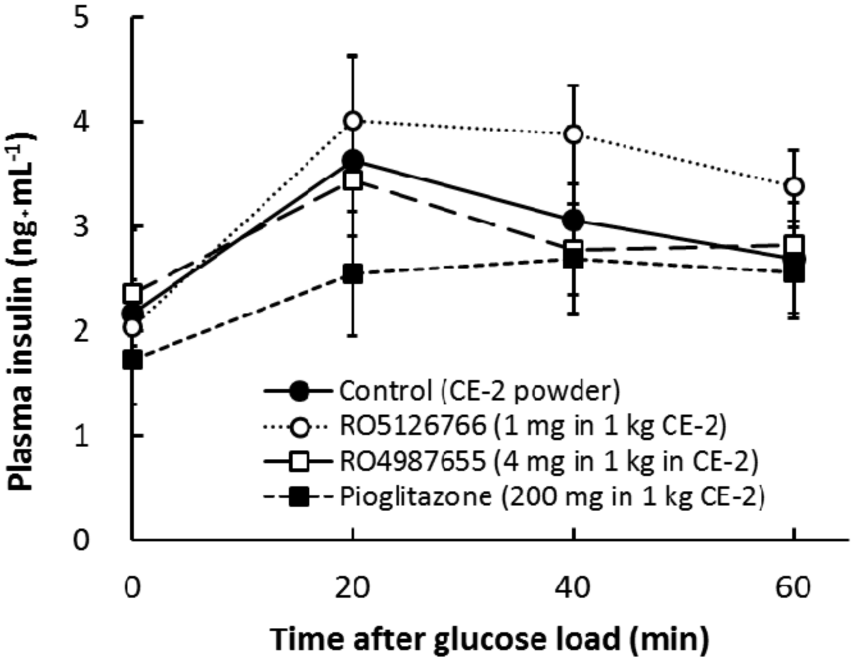

**Supplemental Figure S6**

Effect of RO5126766 (1 mg in 1 kg CE-2), RO4987655 (4 mg in 1 kg CE-2), and pioglitazone (200 mg in 1 kg CE-2) on adipogenic gene expressions in epididymal adipose tissue from *db/db* mice. Epididymal adipose tissue was harvested after treatment for 17 days. Total RNA was extracted using by an RNeasy kit (Qiagen, Limburg, Netherlands) according to the instruction manual. mRNA expression level was determined by quantitative real-time polymerase chain reaction (qRT-PCR) using *Gapdh* as an internal control. Data are expressed as mean ± SEM, $n = 8$. $^{**}p < 0.01$, $^{***}p < 0.001$ *vs.* Control by Dunnett's multiple-comparison test. *Acox1*, acyl-Coenzyme A oxidase 1; *Acsl1*, acyl-CoA synthetase long-chain family member 1; *Fabp4*, fatty acid binding protein 4. All primers were purchased from Applied Biosystems (Carlsbad, CA, USA), Assay ID: *Acox1*, Mm00443579_m1; *Acsl1*, Mm00484217_m1; *Fabp4,* Mm00445880_m1; Part No. of *Gapdh*, 4352339E.

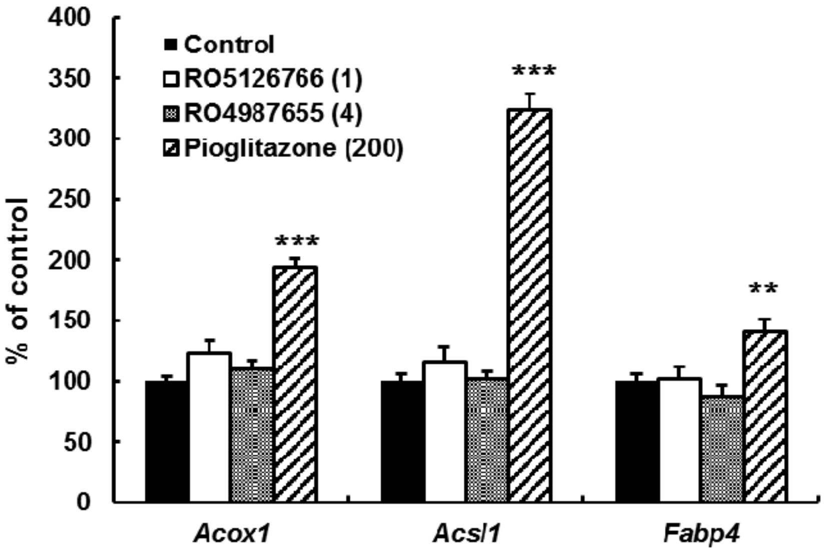

**Supplemental reference**

[1] Nakagawa, T., Tsuchida, A., Itakura, Y., Nonomura, T., Ono, M., Hirota, F., Inoue, T., Nakayama, C., Taiji, M., and Noguchi, H, "Brain-derived neurotrophic factor regulates glucose metabolism by modulating energy balance in diabetic mice", *Diabetes*, vol. 49, no. 3, pp. 436–444, 2000.
